# A randomized controlled trial to prevent childhood obesity through early childhood feeding and parenting guidance: rationale and design of study

**DOI:** 10.1186/1471-2458-13-880

**Published:** 2013-09-24

**Authors:** Elizabeth Reifsnider, David P McCormick, Karen W Cullen, Laura Szalacha, Michael W Moramarco, Abigail Diaz, Lucy Reyna

**Affiliations:** 1 College of Nursing and Health Innovation, Arizona State University, 500 N. 3rd Street, Phoenix, AZ 85004, USA; 2 School of Medicine, University of Texas Medical Branch, 301 University Boulevard, Galveston, TX 77555-1119, USA; 3 USDA/ARS Children’s Nutrition Research Center, Baylor College of Medicine, 1100 Bates Street, Houston, TX 77030, USA; 4 College of Nursing, Ohio State University, 1585 Neil Avenue, Columbus, OH 43210, USA; 5 Houston Department of Health and Human Services, 8000 North Stadium Drive, Houston, TX 77054, USA; 6 Reina Enterprises, 2002 S. Wayside, Houston, TX 77023, USA

**Keywords:** Childhood obesity, Home visiting, Community approach

## Abstract

**Background:**

Early and rapid growth in Infants is strongly associated with early development and persistence of obesity in young children. Substantial research has linked child obesity/overweight to increased risks for serious health outcomes, which include adverse physical, psychological, behavioral, or social consequences.

**Methods/design:**

The goal of this study is to compare the effectiveness of structured Community Health Worker (CHW)- provided home visits, using an intervention created through community-based participatory research, to standard care received through the Special Supplemental Nutrition Program for Women, Infants, and Children (WIC) office visits in preventing the development of overweight (weight/length ≥85th percentile) and obesity (weight/length ≥95th percentile) in infants during their first 3 years of life. One hundred forty pregnant women in their third trimester (30–36 weeks) will be recruited and randomly assigned to the intervention or control group.

**Discussion:**

This study will provide prospective data on the effects of an intervention to prevent childhood obesity in children at high risk for obesity due to ethnicity, income, and maternal body mass index (BMI). It will have wide-ranging applicability and the potential for rapid dissemination through the WIC program, and will demonstrate the effectiveness of a community approach though employing CHWs in preventing obesity during the first 3 years of life. This easy-to-implement obesity prevention intervention can be adapted for many locales and diverse communities and can provide evidence for policy change to influence health throughout life.

**Trial registration:**

Clinical Trials Number: NCT01905072

## Background

Childhood obesity has become a major health concern in nearly every country in the world. While 61% of U.S. adults and almost 12% of U.S. children were overweight in 2001, a decade later, over two-thirds of U.S. adults and almost one-third of U.S. children and adolescents were overweight or obese [1]. Many researchers have noted that obesity has the same association with chronic health conditions as does 20 years of aging, and the cost of obesity exceeds the costs of smoking and drinking for national health care use [2-4]. Adolescent obesity may result in up to 1.5 million life-years lost, with total costs of $294 billion when lost productivity is combined with medical costs [5,6].

As of May 2010, 38.2% of Hispanic children age 2 to 19 were overweight or obese, compared with 31.7% of all children [7]. This increasing exposure to obesity for a longer time has profound implications for chronic diseases associated with obesity, such as type 2 diabetes (T2DM), cardiovascular disease, and all-cause mortality [8-10] that are especially prevalent in the Hispanic population. In fact, risks of developing diabetes by the late teens can be predicted as early as age 6, based on blood pressure, body mass index (BMI), fasting glucose, and insulin and lipid values [11,12].

Hispanics of Mexican American origin are at an increased risk for obesity, particularly among lower socioeconomic levels [13]. Hispanic children have the highest percentage of at-risk status and obesity at both 9 months and 2 years [14]. Among children in the lowest income quintile, 40% were obese by 2 years. We are addressing this major health disparity as the children in our sample are at risk for obesity based on their ethnicity, income, and maternal weight status. The National Council of La Raza reports that one out of two Latino children born in the Year 2000 will develop diabetes [15]. This is especially pertinent to the current proposal, which works exclusively with Mexican (residing in the U.S.) and Mexican-American (MA) families to prevent the development of childhood obesity.

Most researchers now realize that by the time a child is age 5, the prime years for prevention of obesity have passed. By this age, many children have established patterns of eating and activity that are difficult to modify. Infancy and early childhood are now viewed as the prime ages for preventing obesity [16-18] as these are the ages of rapid growth with life-long metabolic and behavioral consequences [19].

We propose a behavioral intervention recommended by the IOM [20] focused on growth monitoring (measurement/feedback to parents at home visits), parenting (cue recognition), early feeding education (breastfeeding support and food introduction), and activity and rest/sleep promotion. A pilot study [19] established that interventions aimed at teaching parents soothing/sleeping techniques and appropriate feeding can promote a lower BMI. Our intervention expands on this study by intervening with a higher-risk population and employing community health workers (CHWs), who can provide a cost-effective approach to addressing the obesity epidemic. The project outcomes can have a major impact on childhood obesity in low-income, MA children through its partnership with the Special Supplemental Nutrition Program for Women, Infants, and Children (WIC) program. These outcomes address child health issues of national significance and can lead the way in promoting community-campus partnerships to improve the health of our nation.

The majority of trials to reduce prevalence of childhood obesity have used school-based interventions, which have had little success [16].

Our study is innovative in that it shifts the current research on childhood obesity prevention that has been primarily school-based into the home, where obesity begins. We are challenging the paradigm of waiting until obesity has developed to begin addressing it.

Moreover, our approach to childhood obesity prevention is innovative in that it provides an evidence-based, culturally competent intervention that individual families can personalize for adoption. The majority of intervention studies on pediatric obesity have been conducted with white, middle-class samples [21], thus providing scant knowledge for intervening with low-income, Hispanic populations. A study with primarily middle-income women (6% Hispanic) utilized interventions that were delivered starting at birth through home visits by research nurses [19]. The group receiving both soothe/sleep technique and solid food introduction had significantly lower weight/length percentiles at 1 year than the groups who only received one intervention. This demonstrates that for the studied population, providing both interventions is the most powerful. Our intervention includes the topics taught above, but we have a much more vulnerable sample, and we employ CHWs as the interveners.

While primary prevention is increasingly seen as the most effective means to address the epidemic of childhood obesity, there have been no published studies of interventions beginning in infancy (a critical period that can predict life-long growth patterns) with our specific population: infants born to MA, overweight/obese WIC clients. Our study is significant in that it will be the first of its kind to use an intervention beginning at birth to prevent childhood obesity in this high-risk population. The result is a culturally relevant intervention that can be delivered through community means and that will provide the best opportunity to prevent adult obesity by targeting behaviors in childhood, with all its severe health and social problems. Prevention is more cost effective than treatment and will result in a healthier population [5].

## Methods/Design

The current study is a randomized childhood obesity intervention study of newborn children and their mothers. The study is being conducted with the approval of three appropriate ethics committees and is in compliance with the Helsinki Declaration. The study has been approved by the Arizona State University Institutional Review Board (IRB), the Texas Department of State Health Services IRB, and the City of Houston Health and Human Services research committee. We use a randomized two-group design (Intervention Group [IG], Control Group [CG]) with each group comprising 70 pregnant women who are in their third trimester when they are enrolled from a WIC clinic. The IG will receive the full intervention delivered by CHWs through home visits, with backup from public health nurses. The CG will receive only measurement visits, with no intervention delivered during the home visits.

### Hypotheses

**Hypothesis 1:** Children in the intervention group will remain within their growth centiles in height/weight and weight for age, while children in the control group will increase in height/weight, and weight percentiles more rapidly (> .67 SD) during the first year of life.

**Hypothesis 2:** Fewer children who receive the intervention will have BMI >95th percentile at ages 2 and 3 than the children in the control group.

**Hypothesis 3:** Children who receive the intervention will exclusively breastfeed for a longer period of time than will children in the control group.

**Hypothesis 4:** Children who receive the intervention will have a higher percentage of fruits and vegetables and a lower percentage of sweetened beverages, desserts, and candy in their diets at ages 1 and 2 than will children in the control group.

**Hypothesis 5:** Parents in the intervention group will be more responsive to infant feeding cues (hunger, satiety) than parents in the control group.

### Aim of the study

The specific aim of this study is to compare the effectiveness of structured CHW- provided home visits, using an intervention created through community-based participatory research, to standard care received through WIC office visits in preventing the development of overweight (weight/length ≥85th percentile) and obesity (weight/length ≥95th percentile) in infants during their first 2 years of life.

### Study criteria and follow-up

#### Maternal inclusion criteria

Mothers that meet the following criteria are eligible for inclusion in the study: Self-described as Mexican or Mexican-American (MA), pre-pregnant BMI of 25 or greater, speaking English or Spanish, between the ages of 18 and 40, living in a home where she can receive home visitors, has no diagnosed chronic diseases that can affect the growth of a fetus (cardiac, respiratory, etc.), has a telephone contact, and is not intending to move from the area. Gestational diabetes is be noted but is not an exclusion criterion; however, Type 1 diabetics are excluded. Exposure to tobacco smoke (either maternal or household smoking) is noted as it is associated with infant obesity, but is not be an inclusion or exclusion criteria.

#### Infant inclusion criteria

We will enroll the full-term singleton infants of enrolled mothers. The infants will be enrolled if they are ≥ 38 weeks gestation, have a birth weight ≥ 2500 gm, and are without endocrine/chromosomal/genetic abnormality that could interfere with growth.

#### Maternal exclusion criteria

Exclusionary criteria for mothers are: Not enrolled in study WIC clinic or does not plan to continue with the study WIC clinic after delivery, high-risk pregnancy, hospitalized after discharge of infant, separated from infant, or those who experienced significant postpartum complications. If these criteria develop in an enrolled mother, she will remain in the study per CONSORT guidelines [22], but her infant will not be followed for study outcomes. We have sufficient power with our sample size to allow for these events.

#### Infant exclusion criteria

Exclusionary criteria for infants are: not discharged home with the mother or who are otherwise separated from their mothers, or who have a severe illness that can affect growth. CONSORT guidelines will apply in these cases as well as in maternal cases.

#### Participant recruitment

Recruitment occurs at a WIC clinic sponsored by the Houston Department of Health and Human Services. The clinic is located in an older area of the city and is heavily Hispanic and low income. The Community census tracts are among the lowest of all census tracts in Harris County (the county in which Houston, TX is located) for the Child Well-Being Index. Sixty-nine percent of residents live below 200% of the Federal Poverty Index, and 51% of area residents have less than a high school education, almost twice the percentage of Houston residents. Sixty-three percent of all births in the area are to mothers who do not have a high school diploma, compared with 36% for Harris County. Infant mortality is 7.2 deaths/1,000 live births, compared with Houston’s 6.5. Houston’s population is over 4 million, and the catchment area for selected WIC clinic serves nearly 500,000 residents. The WIC clinic provided services to 7667 pregnant women in 2012. The maternity clinic at the same site as the WIC clinic delivered 605 women in 2010, approximately 70% of whom were overweight at the time of conception with the majority of those women also receiving WIC at the selected clinic. We recruit pregnant women in their third trimester from the WIC clinic. We have access to a private room for participant recruitment. We enroll the pregnant women at their homes into the study during the prenatal home visit and we obtain written informed consent for themselves and their infants to participate in the study, when they are delivered. The women are not randomized into the intervention or control group until the initial prenatal visit is completed so the data collector will remain blinded to study assignment. We enroll the infants of the study mothers into the study at the time of the 1-week postpartum home visit. Based on the number of pregnant women receiving care at the WIC clinic selected for the proposed study, the fact that the recruiters are Spanish-speaking CHWs with experience working with MA women, and our previous experience conducting similar studies, we do not anticipate any difficulty recruiting an adequate sample size of 150 mothers for this study (75 per group).

### Study groups

#### Intervention group

CHWs deliver the intervention in the participants’ homes. Home visits are arranged at participants’ convenience and occur on a planned schedule: at 36 weeks of pregnancy; at 3 days after birth; at 2 weeks of age; and at 2, 4, 6, 9, 12, 18, and 24 months. This schedule of visits matches the recommended schedule from American Academy of Pediatrics for well-child visits and is timed to coincide with changes in infant feeding and associated nutrition counseling. The CHWs are bilingual in English and Spanish and provide the intervention content using colorful images and simple text; print materials with key concepts are also given to the participant at the end of each visit to aid recall. All materials are bilingual (English and Spanish) and at a 4th-grade reading level or primarily pictorial to avoid literacy issues.

The intervention content is based on the IOM recommendations: [20]

1. Growth monitoring: weight/length taken and graphed at each home visit to show the mother how the infant is growing and to monitor for rapid weight gain.

2. Feeding: support exclusive breastfeeding until 6 months and continue as long as mother/baby desire; delay solid feeding until 6 months; appropriate amounts of food for age; stop bottle feeding at 12 months; have nothing but breast milk/formula/4 oz juice in bottle; limit juice amount to 4 oz per day; introduce cup by 10–11 months; no sweetened beverages; limited amounts of sweets.

3. Parenting: recognizing hunger and satiety cues; handling colic/crying; engaging baby in play.

4. Activity: being active with the baby; no screen time for baby and limited to 1 hour for 1–3 year olds; promote active play while maintaining safety.

5. Sleep: at least 10–12 hours sleep per day needed; promoting sleeping environment for baby.

In addition to these intervention visits, they also receive measurement visits from a bilingual research assistant (RA) who is blinded to the intervention or control status of the participants she visits. All infant and maternal measures (see Table 1) are collected for baseline at 36 gestational weeks, 1 week of the child’s life during the first postpartum home visit when the infant is enrolled in the study, and then at 1, 6, 12, 18, 24, and 36 months, totaling 8 visits. The participants also receive a monthly phone call, beginning at one month after the child’s birth, for a brief survey of their breastfeeding status. These phone calls will last for 12 months or until the mother ceases breastfeeding her child.

**Table 1:** Research measures

**Ecological level**	**Purpose of measure**	**Variable**	**Measure**	**Instruments**	**When/*How *collected**
**Child**	Study Outcome	Anthropometrics	Length, weight W/L or BMI graph	Scale/stadiometer	1 week, 1, 6, 12, 18, 24, 36 months *Physical measure*
	Study covariate, intervention component	Sleep	Hours of infant sleep	BISQ	1, 6, 12, 18, 24, 36 months *Questionnaire*
	Study covariate, intervention component	Screen time	Home Observation Measurement Environment	*HOME tool*	1 week, 1, 6, 12, 18, 24, 36 months *Observation*
	Study outcome, intervention component	Age of food introduction	Week when solid food introduced	24-hr diet recall, analyzed with NDSR; Questionnaire	1, 6, 12, 18, 24, 36 months *Interview*
**Food**	Study outcome, intervention component	Daily food intake	Fruit, vegetables, sweet drinks, desserts, candy		*questions about breast/bottle feeding will cease when child weaned.*
	Study outcome, intervention component	Breast/Bottle Feeding	Amt. formula/times Breastfed per day	Telephone Questionnaire	Monthly for 12 months after birth or until breastfeeding ceases
**Parent/Home**	Study outcome, intervention component	Parent–child interaction	Feeding scale Teaching scale	FITS	1 week, 1, 6, 12, 18, 24, 36 months *Observation*
	Study covariate, intervention component	Maternal size	Pre-pregnant BMI	Scale/stadiometer	From Prenatal WIC record *Existing data*
	Study covariate	Age, education, etc.	Demographics	Questionnaire	Prenatal record *Existing data*
	Study covariate	Acculturation	Brief ARSMA	Questionnaire	Prenatal *Questionnaire*

#### Control group

The CG receive only the 8 measurement visits from the RA and the monthly breastfeeding survey calls. All other care and resources for the participants in the CG will be provided by the WIC clinic, should they choose to use them.

### Measurements

#### Anthropometrics

Length is measured with portable recumbent length board to the nearest mm, and weight is measured to the nearest 0.1 kg using an electronic digital portable scale with the infant/child wearing only a dry diaper. The child will be measured recumbent until 2 years of age to maintain consistency in measurements. The length and weight measures are recorded on the Weight for Length (W/L) percentile WHO growth grids as recommended by the Centers for Disease Control and Prevention (CDC)^87^ to determine body mass in children under age 2. In the WHO charts, the healthy breastfed infant is the standard against all other infants are compared. When the child is 2 years of age, we will use standing height and weight to calculate BMI, which we will enter on the 2–5 year old BMI grids provided by the CDC to determine BMI percentiles for children [23]. For infants (birth to 24 months) and children (2 to 3 years), the W/L percentile and the BMI measure respectively will be converted to a z-score to allow group comparison by sex and age in months. The infant’s W/L is measured, graphed, and shown to parents at each home visit to the IG to show the child’s growth trajectory. These CHW-obtained measurements are not used as outcome criteria but as feedback for parents.

#### Sleep

Sleep is assessed with the Brief Infant Sleep Questionnaire (BISQ) [24,25], a 20-item questionnaire that assesses bedtime problems, excessive sleepiness, awakenings, regularity of sleep, and sleep-disordered breathing. The questionnaire is used for, and includes questions about, infants, toddlers, and preschool children and has been validated with significant correlations with actigraphy and sleep diaries.

#### Screen time

The Home Observation for Measurement of the Environment (HOME) Inventory [26,27] is used to measure the child’s early developmental environment in his/her home. The HOME includes a measure of total hours a day the television is on in the home, and the total hours per day the child watches television. The mother is also asked for hours of other screen time (computer, video played on the television, etc.) per day. The HOME is administered in the family home, through casual dialogue and careful observation by a trained observer. The CHWs and the measurement RA have been trained in the HOME by the PI, who has received training for teaching the HOME.

#### Nutrition

The Feeding Infants and Toddlers Study (FITS) is the guiding framework for the dietary data collection and analysis [28-31]. The FITS is a cross-sectional descriptive survey of a national random sample of US children from birth through age 3 years and reports in detail the dietary intakes of infants and young children. The FITS used the 24-hour diet recall with follow-up questions, and these methods will guide our data collection and analysis. The recall captures all food intake for the 24 hours of measurement, both food consumed at home and food consumed away from home. Parents/guardians are most involved in preparing meals for their children and feeding their children [32,33] and are able to provide a 24-Hour Diet Recall for their children’s diets.

#### Demographics

Parental age, education level, employment status, job title, etc. are obtained from the prenatal record and if not present, are queried from each participant. Childcare patterns are included if the parents are employed outside the home. The number of hours the child is in day care, the type of day care provider, and how the child is fed at the day care provider’s facility are assessed along with the food available through day care. Information on public assistance, such as Medicaid, Temporary Assistance for Needy Families (TANF), and Food Stamps, are also obtained to determine the family’s income status. These data are used to describe the sample characteristics.

#### Acculturation

The Brief Acculturation Rating Scale for Mexican Americans-II (Brief ARSMA-II) [34,35] measures acculturation using variables of language, ethnic identity, and ethnic interaction. The Brief ARSMA-II is able to generate both linear acculturation categories (Levels 1–5) and orthogonal acculturative categories (Traditional, Low Bicultural, High Bicultural, and Assimilated). Higher total scores indicate that participants are acculturated into the Anglo-American culture.

### Data management

Measurement data are collected only by the RA. The 24-hour diet recall is done through an online system known as ASA24™ [36]. All other data are collected online through REDCap™, a web-based electronic data capture tool [37]. Data are collected at 20 different points for each mother-child dyad. A total of 10 instruments, surveys, and questionnaires are used at various times throughout the project, and REDCap™ was used to create 9 of the 10 instruments, excluding a 24-hour diet recall. All of the data collected from these instruments will be analyzed in SPSS, and REDCap’s™ data export function allows for direct export of all data into the proper format.

REDCap™ allows researchers to design or download secure online forms for data collection, management, and analysis. The easy-to-use interface and data management and analysis capabilities of the program make it an ideal tool for use in longitudinal studies, studies with researchers in diverse locations, and studies with multiple instruments. After completion of the data collection forms, and the start of actual data collection, data management and monitoring is secure and simple. The real-time nature of the web-based electronic data capture system allows for investigators and data managers to observe from afar as all data is entered. The data collection interface also minimizes user error through a set system of checks and an easily read data collection table.

## Discussion

Preventing obesity in infants and young children is a promising approach to reversing the childhood and adult obesity epidemic as childhood obesity tends to persist into adulthood and increases the risk of cardio-metabolic diseases. The patterns of eating, physical activity, and sleep are developed in childhood, patterns that continue to influence obesity, health, and quality of life throughout life [20]. Our proposed study will provide prospective data on the effects of an intervention to prevent childhood obesity in children at high risk for obesity due to ethnicity, income, and maternal BMI. Our study has broad and wide-ranging applicability and the potential for rapid dissemination through WIC. We hope to demonstrate the effectiveness of a common community approach (Community Health Workers) in preventing obesity during the first 2 years of life as well as the intervention’s persistence from age 2 to 3. This easy-to-implement obesity prevention intervention can be adapted for many locales and diverse communities and can provide evidence for policy change to influence health throughout life.

## Competing interests

The authors declare that they have no competing interests.

## Authors’ contributions

ER and DPM conceived the study and developed the protocol with input from KWC and LS and all four submitted the proposal for funding. MM, AD, and LC substantially contributed to the operationalization of the study once funded. All the authors contributed to the drafting of the manuscript. All the authors have read and approved the final manuscript.

## Pre-publication history

The pre-publication history for this paper can be accessed here:

http://www.biomedcentral.com/1471-2458/13/880/prepub
